# Efficacy of Hair Mesotherapy With Biomimetic Peptides and Micronutrients in Patients With Androgenetic Alopecia: A Retrospective Observational Study

**DOI:** 10.1111/jocd.70993

**Published:** 2026-06-19

**Authors:** Özlem Köse, Murat Borlu

**Affiliations:** ^1^ Department of Dermatology, Faculty of Medicine İstanbul Aydın University İstanbul Turkey; ^2^ Department of Dermatology, Faculty of Medicine Erciyes University Kayseri Turkey

**Keywords:** androgenetic alopecia, biomimetic peptides, hair loss, mesotherapy, micronutrients

## Abstract

**Background:**

Hair mesotherapy is increasingly used in the management of androgenetic alopecia (AGA), yet standardized treatment protocols and controlled clinical data are limited.

**Aims:**

This study aimed to evaluate the efficacy and safety of a mesotherapy formulation containing biomimetic peptides, vitamins, and micronutrients in patients with AGA.

**Patients/Methods:**

We conducted a retrospective observational study including 50 patients clinically diagnosed with AGA. Mesotherapy was administered using a mesogun for eight sessions over 3 months. Each session involved 3 mL of mesotherapy solution containing biomimetic peptides, vitamins, and micronutrients. Clinical, trichoscopic, and Trichoscan assessments were performed before treatment and 4 weeks after the final session.

**Results:**

Post‐treatment, significant improvements were observed in trichoscopic parameters, including reduced hair diameter variability (*p* < 0.01), decreased empty follicles (*p* = 0.012), and increased upright vellus hairs (*p* < 0.01). Trichoscan analysis demonstrated significant increases in total hair count, hair density, average hair thickness, terminal hair density, and terminal hair ratio (all *p* < 0.001). Treatment was well‐tolerated, with only mild, transient pain reported.

**Conclusions:**

Hair mesotherapy with biomimetic peptides and micronutrients appears to be a promising and well‐tolerated therapeutic option for AGA. Larger prospective controlled trials are needed to validate these findings.

**Abbreviations**
AGA: androgenetic alopecia
FAGA: female androgenetic alopecia
FGF: fibroblast growth factor
IGF: insulin‐like growth factor
KGF: keratinocyte growth factor
PRP: platelet rich plasma
TE: telogen effluvium
VEGF: vascular endothelial growth factor
Vit: vitamin

## 1 Introduction

Androgenetic alopecia (AGA) is the most common form of hair loss, affecting approximately 50% of men and women during their lifetime [1]. Current therapeutic options include topical minoxidil, oral finasteride, low‐dose oral minoxidil, spironolactone, cyproterone acetate, platelet‐rich plasma (PRP), microneedling, mesotherapy, and low‐level laser therapy. Although some of these treatments are effective, many are limited by systemic side effects, variable response rates, or insufficient long‐term data [1, 2].

Mesotherapy is a minimally invasive technique that delivers active agents directly into the dermis and has been used in dermatology for over six decades [3]. In hair disorders, mesotherapy has been applied for AGA, telogen effluvium (TE), alopecia areata, chemotherapy‐induced alopecia, and cicatricial alopecia. However, treatment protocols remain highly heterogeneous, with differences in ingredients, dosages, and session schedules, making it difficult to compare studies and establish standardized guidelines [3, 4].

Commonly used mesotherapy agents for hair loss include minoxidil, dutasteride, growth factors, biomimetic peptides, vitamins, and micronutrients [5, 6]. Among these, biomimetic peptides have attracted attention due to their ability to mimic the activity of naturally occurring growth factors such as vascular endothelial growth factor (VEGF), insulin‐like growth factor‐1 (IGF‐1), and fibroblast growth factor (FGF), all of which play critical roles in hair follicle cycling and angiogenesis [6, 7, 8, 9].

This study aimed to evaluate the clinical efficacy and safety of a mesotherapy formulation containing biomimetic peptides, vitamins, and micronutrients in patients with AGA through a retrospective observational analysis.

## 2 Materials and Methods

### 2.1 Study Design and Patients

This retrospective study included patients diagnosed clinically with AGA who were presented to our dermatology department between April 2021 and June 2025. Ethical committee approval was received. Informed consent was obtained from all patients. All the patients were instructed about the alternative treatment methods including minoxidil and finasteride.

Patient demographics (age, gender, and skin phototype) were recorded, and AGA severity was classified using the Ludwig and Hamilton scales. We excluded patients with abnormal laboratory results (ferritin, ANA, VDRL, vitamin (Vit) B12, and folate), a positive hair pull test, or those who had received systemic or topical treatments (including devices such as lasers or microneedling) within the past year.

Clinical and trichoscopic photographs were obtained using the MoleMax device (Derma Medical Systems, Vienna, Austria). Trichoscopic parameters included hair diameter variability, peripilar halo, empty follicles, and upright vellus hairs. Images were taken at ×30 magnification from marked scalp regions. Trichoscan analysis was performed at baseline and 4 weeks after treatment completion.

Patients with Ludwig‐pattern hair loss were photographed with a videodermoscope from three midline locations: anterior, middle, and posterior, and the Trichoscan results from these three locations were averaged. For those with Hamilton‐pattern hair loss, the Trichoscan measurements from three locations were averaged, including the two symmetrical frontal locations 1 cm inside the hairline and vertex. Total hair count, hair density, hair mass, hair thickness means, density of terminal hairs, and ratio of terminal hairs were recorded before the first session of mesotherapy.

Each session involved 3 mL of solution: 1.5 mL DermaHeal HL and 1.5 mL DermaHeal Stem C'rum HL (Caregen Co. Ltd., South Korea). Ingredients for DermaHeal HL: sh‐Oligopeptide‐2 (Insulin like growth factor‐1: IGF‐1) 1 mg, sh‐Polypeptide‐1 (b‐ FGF) 1 mg, sh‐Polypeptide‐9 (VEGF) 2 mg, Copper Tripeptide‐1 10 mg, Coenzyme‐A 2.5 mg, Nicotinamide adenine dinucleotide 7 mg, Disodium flavin adenine dinucleotide 1 mg, Vit A 0.25 mg, Vit B1 0.25 mg, Vit B2 0.25 mg, Vit B3 10 mg, Vit B5 0.025 mg, Vit B6 0.025 mg, Vit B7 0.025 mg, Vit B12 0.25 mg, Vit C 50 mg, Vit E 0.025 mg, Vit I 0.125 mg, Vit K 0.025 mg, folic acid 0.025 mg, aminobutyric acid 60 mg, alanine 32 mg, glycine 14 mg, isoleucine 18 mg, methionine 4 mg, proline 6 mg, threonine 19 mg, valine 25 mg, aspartic acid 10 mg, arginine 10 mg, cystine 10 mg, hydroxyproline 4 mg, leucine 20 mg, ornithine 7 mg, serine 11 mg, tryptophan 18 mg, glutamic acid 8 mg, asparagine 8 mg, glutamine 136 mg, histidine 20 mg, lysine 31 mg, phenylalanine 17 mg, taurine 4 mg, tyrosine 16 mg, calcium chloride 200 mg, potassium chloride 400 mg, magnesium sulfate 100 mg, sodium phosphate 6600 mg, adenosine cyclic phosphate 10 mg, cytosine 10 mg, guanosine 10 mg, thymine 10 mg, glutathione 10 mg. All concentrations were milligrams in liters within 5 mL vials. Ingredients for Dermaheal Stem C'rum HL: sodium chloride, biotin, copper tripeptide‐1, sh‐oligopeptide‐2 (CG‐IGF‐1), sh‐polypeptide‐1 (CG‐bFGF), and sh‐polypeptide‐9 (CG‐VEGF) within 100 mg powder and 5 mL (sodium chloride and water) vials were mixed. The injections were spaced 0.5–1 cm apart.

Treatments were administered with a mesogun (Pistor Eliance, France) using 30G, 13 mm needles at a depth of 2 mm. Multiple punctures were made by the device in a continuous mode for the entire alopecic area. Every session lasted 15–20 min. Patients were informed not to wash their scalp for at least 24 h. Patients underwent four weekly sessions followed by four biweekly sessions, for a total of eight sessions over 3 months. After completing the 3‐month treatment protocol, patients were photographed both clinically and with trichoscopy 4 weeks after the last session. All the clinical, trichoscopic, and Trichoscan records were re‐evaluated 4 weeks after the end of the therapy. This paper is a retrospective, observational, and clinical study.

Patients who used any other type of treatment for AGA within a 1‐year period were excluded from the study. Patients with laboratory anomalies which may cause TE within the postpartum period and with a positive light pull test were also excluded.

All statistical analyses were performed using appropriate software (SPSS version 22, IBM Corp., Armonk, NY, USA). Continuous variables were expressed as mean ± standard deviation (SD), and categorical variables as counts and percentages.

For parametric continuous variables, comparisons between baseline and post‐treatment values were performed using the paired sample *t*‐test. For nonparametric categorical variables, changes before and after treatment were assessed using the McNemar test. A *p*‐value of < 0.05 was considered statistically significant.

## 3 Results

### 3.1 Patient Characteristics

Fifty patients (39 women, 11 men) with a mean age of 47.7 ± 14.8 years (range: 24–91) were included. Skin phototype distribution was Type II (14 patients), Type III (26 patients), and Type IV (10 patients). AGA severity distribution was: Ludwig Type I (*n* = 14), Type II (*n* = 18), and Type III (*n* = 5); Hamilton Type II (*n* = 3), Type III (*n* = 4), Type IV (*n* = 5), and Type V (*n* = 1).

### 3.2 Trichoscopic Results

**FIGURE 1:**
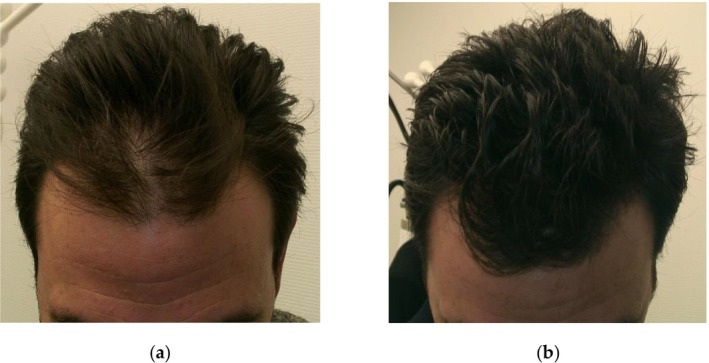
Clinic photos before (a) and after (b) hair mesotherapy.

**FIGURE 2:**
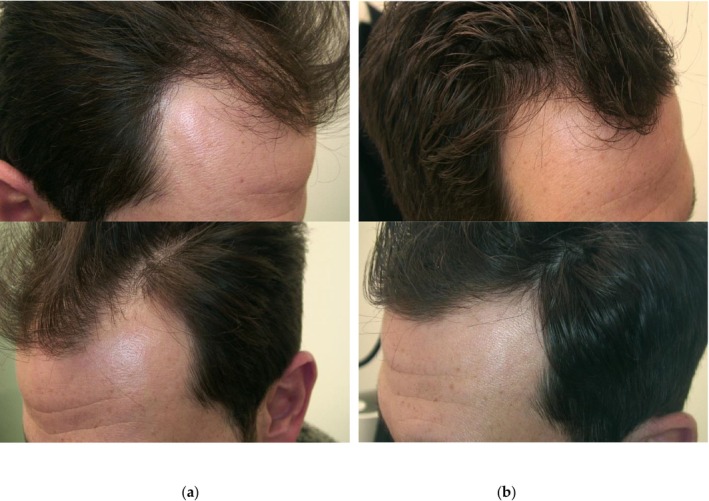
Clinic photos before (a) and after (b) hair mesotherapy.

**FIGURE 3:**
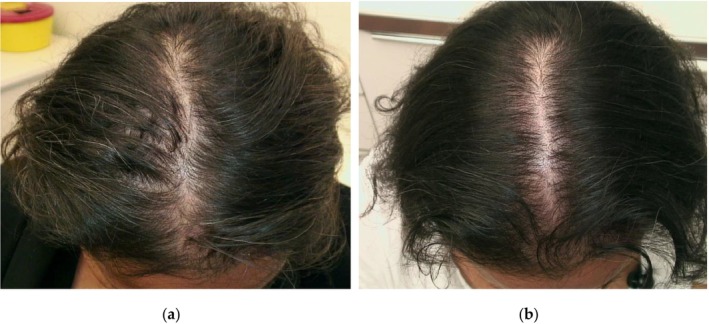
Clinic photos before (a) and after (b) hair mesotherapy.

**FIGURE 4:**
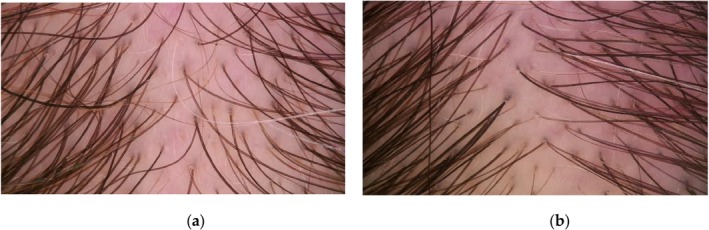
Representative trichoscopic images before (a) and after (b) hair mesotherapy. (a) Increased hair diameter variability and empty follicles before treatment; (b) Decreased variability and fewer empty follicles after treatment.

**FIGURE 5:**
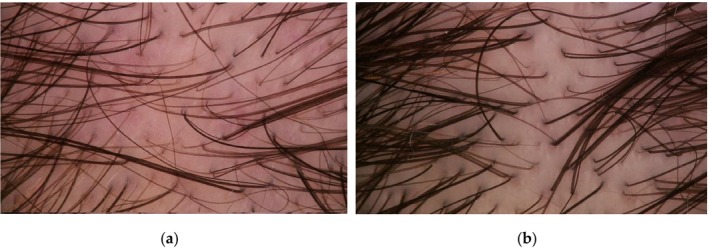
Representative trichoscopic images before (a) and after (b) hair mesotherapy. (a) Increased hair diameter variability and empty follicles before treatment; (b) Decreased variability after treatment.

Clinical photos of the patients before and after hair mesotherapy are below (Figures 1, 2, 3). After treatment, hair diameter variability, and empty follicles were significantly reduced (*p* < 0.01 and *p* = 0.012, respectively). Upright vellus hairs significantly increased (*p* < 0.01). No significant changes were observed in peripilar halo or yellow dots (Table 1; Figures 4 and 5).

**TABLE 1:** Comparison of trichoscopic features before and after hair mesotherapy in patients with androgenetic alopecias.

Trichoscopy results	Patients with androgenetic alopecia (*n* = 50)		
	Before treatment	After treatment	*p*
Hair diameter variability	**50**	**35**	**< 0.01**
Empty follicles	**22**	**10**	**0.012**
Peripilar halo	21	21	1
Yellow dots	13	8	0.227
Upright vellus hairs	**0**	**20**	**< 0.01**

*Note:* Values represent the number of patients presenting the respective feature. Statistical significance was determined using paired tests; *p* < 0.05 was considered significant. The before and after treatment values corresponding to bold *p* values are re‐written as bold characters. In section 4.6 (Trichoscopy and trichoscan of AGA) all the significant bold values are discussed in detail.

### 3.3 Trichoscan Results

All trichoscan parameters showed significant improvement after treatment (*p* < 0.001) (Table 2).

**TABLE 2:** Trichoscan results before and after hair mesotherapy in patients with androgenetic alopecia.

Trichoscan results	Patients with androgenetic alopecia (*n* = 50)		
	Before treatment	After treatment	*p*
Total hair count	72.9 ± 24.8	87.5 ± 27.9	< 0.001
Hair density	213.7 ± 114.8 1/cm^2^	236.6 ± 77.8 1/cm^2^	< 0.001
Average hair thickness	52.7 ± 8.4 μm	55.5 ± 8.9 μm	< 0.001
Terminal hair density	128.7 ± 56.8 1/cm^2^	159.2 ± 58.6 1/cm^2^	< 0.001
Terminal hair ratio	61% ± 13.4%	68.6% ± 13.8%	< 0.001

*Note:* Values expressed as mean ± standard deviation. Statistical significance was determined using paired tests; *p* < 0.05 was considered significant.

### 3.4 Adverse Effects

The treatment was well‐tolerated. The only side effect reported was mild transient pain during injections, which resolved within an hour.

### 3.5 Limitations

Our study has a retrospective design. The absence of a control group and the relatively short follow‐up period were the limitations. Outcome assessments were performed by both observers together at the same time.

## 4 Discussion

### 4.1 Hair Mesotherapy Studies in Literature

Mesococtails and mesoproducts are widely used for hair mesotherapy in the field of aesthetic dermatology [1]. One of the advantages of mesotherapy is that it enables the penetration of large molecules into the subcutaneous layer. Compared to the topical application, it can yield faster results, require lower doses, and is believed to deliver outcomes more quickly [1]. In this study, the reason for using the mesogun is that the application is faster, more consistent, and easier. Another advantage of mesotherapy and mesogun is thought to be their ability to increase microcirculation, which may stimulate regenerating cytokines and growth factors and trigger new tissue formation due to the creation of numerous microchannels in the skin [1, 2]. In this study, mesotherapy was effective solely without any systemic or topical treatment. Shome et al. [3] showed that intradermal injections of mesotherapy products were established as slightly more efficacious than the derma roller technique. We also preferred to solely inject the products with a mesogun instead of applying a derma roller.

The purpose of hair mesotherapy is documented to improve hair quality, moisturize the scalp, extend the anagen phase, inhibit 5‐α reductase, enhance microcirculation, and provide nutrient support [1]. Although there are many mesotherapy products used for various types of hair loss, the number of controlled studies is limited. Upon reviewing the studies, it has been observed that these products contain minoxidil, finasteride, dutasteride, saw palmetto, biotin, panthenol, hyaluronic acid, taurine, and multivitamins [1]. The number of publications in the literature reporting that low‐dose oral minoxidil is effective in the treatment of AGA is steadily increasing. The purpose of applying minoxidil as a mesotherapy, as with other hair mesotherapy products, is to enhance local bioavailability while avoiding the systemic side effects of it [4]. Uzel et al. [5] injected 0.5% minoxidil solution once a week for 10 weeks and demonstrated that it was an effective treatment for female androgenetic alopecia (FAGA). A comparative study between 2% minoxidil topical spray vs. intradermal injection mesotherapy with minoxidil has defined that minoxidil injection is more effective than using it as a topical application for FAGA [2]. This study also used a mesogun for the intradermal injections in the scalp [2]. In three different randomized studies, mesotherapy with dutasteride was found to be effective compared to placebo [6, 7, 8]. However, D‐panthenol, biotin, and pyridoxine were also present in the ingredients in all three studies. The combination of these ingredients with dutasteride was reported to be more effective than dutasteride alone [7]. In our study, we used the same molecules except minoxidil [6].

Studies with minoxidil and dutasteride injections encountered some side effects, including injection site infections, granulomatous foreign body reactions, fat necrosis, lichenoid drug eruptions, Nicolau syndrome, pain, headache, itching, frontal edema, facial swelling, and alopecia [9, 10, 11]. Our patients did not complain of any of the side effects mentioned above.

### 4.2 Hair Mesotherapy With Biomimetic Peptides

Growth factors that stimulate different stages of the hair cycle [12]. They include VEGF, epidermal growth factor, IGF‐1, FGF, wingless related integration site, noggin, keratinocyte growth factor (KGF), and copper tripeptides [13, 14]. PRP stands out due to its regenerative properties in hair loss and the successful outcomes demonstrated in the literature [15]. PRP is prepared by taking the patient's own blood and contains a variety of growth factors, primarily VEGF, FGF‐7, IGF, and PDGF [16]. FGF has been shown to prolong the anagen phase of the hair cycle [16]. VEGF has also been shown to play a significant role in blood flow to hair follicles and to increase density. IGF‐1 prevents apoptosis in hair follicle cells [16]. PDGF and KGF (FGF‐7) support hair follicle regeneration by promoting epithelial cell proliferation and follicular differentiation [16]. Research suggests that the mechanism of action of PRP in the treatment of alopecia primarily operates through growth factors [17]. Gold et al. [18] revealed that hair mesotherapy, including biomimetic peptides, ensures a better treatment option for regrowth after hair transplantation.

A first‐in‐man pilot clinical study described that these hair growth factors, when used in combination (VEGF, bFGF, IGF, KGF, thymosin β4, and copper tripeptide‐1), have a synergistic impact on hair growth [19]. In bioengineered recombinant formulations, specialized molecules that exhibit effects similar to growth factors are developed and are referred to as biomimetic peptides due to their comparable effects with PRP [13]. In our study, products which contain biomimetic peptides, vitamins, and micronutrients were used. The most extensively studied other brand with similar ingredients, including growth factors and biomimetic peptides, is QR (Quick Response) 678 and QR 678 Neo [13]. In an in vitro study, Sh‐Polypeptide 9 (CG‐VEGF), a VEGF‐like synthetic biomimetic peptide, promoted VEGF production efficiently and stimulated the production of β‐catenin, an intracellular signaling molecule that takes a role in the regulation of endothelial and hair follicle dermal papilla cells [20]. The Sh‐Polypeptide 9 (CG‐VEGF) is also one of the ingredients that is used in this study. It is demonstrated that the Wnt/β‐catenin pathway is critically important for the continuation of the hair follicle cycle and is thought to have a potential role in AGA treatments [21]. Dermal papilla cells have been reported to synthesize FGF and IGF‐1, facilitating the transition from the telogen to the anagen phase [22]. Additionally, FGF7 and FGF10 have been shown to play a significant role in the activation of epithelial stem cells [23]. IGF‐1 has been demonstrated to trigger the anagen phase for healthy hair formation, prevent apoptosis, and increase VEGF [21]. Synergistic effects of FGFs and IGF‐1 are necessary for the activation of epithelial stem cells in the bulge region from a quiescent state [21]. Targeting dermal papilla cells with these growth factors has been reported to potentially reduce hair follicle miniaturization and decrease hair growth in patients with AGA [24]. Also, intradermal injections of the combinations of growth factors proved to be a successful treatment of FAGA in patients with PCOS [25]. We used biomimetic peptides to simulate the effects of VEGF, FGF and IGF in this study, and our results were promising.

Literature regarding the combination of treatments with hair mesotherapy is scarce. There is a study that proved the biomimetic peptides hair mesotherapy is beneficial, but combination therapy of biomimetic peptides hair mesotherapy with minoxidil 5% solution and 1 mg finasteride may produce better results [26]. Further studies investigating combination therapies are necessary.

### 4.3 Hair Mesotherapy With Micronutrients

Gajjar et al. [27] proposed that hair mesotherapy containing 56 constituents, which included 24 amino acids, 13 vitamins, four coenzymes, four nucleic acids, five minerals, two reducing agents, decapeptide 4, acetyl decapeptide, and copper tripeptide, was as efficient as topical 5% minoxidil. In our study, we achieved successful results using a formulation similar to that of Gajjar et al. [27], but with the addition of biomimetic peptides. A similar randomized controlled study comparing the results of mesotherapy with vitamin‐minerals alone and topical minoxidil with 30 patients declared more successful results with the mesotherapy group [28]. Multivitamin mesotherapy containing vitamin A, B, C, D, E; dexpanthenol; saw palmetto; gingko biloba; cysteine; methionine; taurine; biotin; caffeine; minerals; and zinc improves local microcirculation and, when combined with needling, promotes restoration in cases of hair loss [29]. An in vivo study revealed dexpanthenol (Vit B5) modulates gene expressions of hair keratin associated proteins and plays a crucial role in forming strong hair shafts [30]. Vitamin B5 was also an ingredient in our study. Hair mesotherapy including minoxidil, finasteride, biotin, and d‐panthenol was reported as an excellent response in male AGA [31]. In this study, the mesotherapy product also contained a multitude of vitamins and micronutrients, and we encountered encouraging results. The efficacy of the polydeoxyribonucleotide injections in treating FAGA was reported as efficient [32]. In our study, the mesotherapy product also contained dinucleotides, which may have additional effects for hair growth. Deficiencies of iron, Vit D, folate, Vit B12, and selenium are linked to premature hair graying/whitening in early adulthood [33]. Some of our patients also reported a reduction in the graying of their hair after the treatment, but we did not conduct any measurements related to this.

### 4.4 Adverse Effects

A cellular toxicity and animal efficacy study of these formulations showed that they are safe with no cellular toxicity [13]. Also, hair mesotherapy treatment was well‐tolerated in the other reports [19, 34]. Mild pain, burning sensation, hair loss including cicatricial alopecia, frontal edema, multifocal abscess at the injection site of mesotherapy, local irritation, allergic reactions, infections, and melanoma on the scalp after anti‐hair loss mesotherapy with growth factors have been reported [10]. Our patients have no complaints about the procedure except the mild pain during the injections, which completely disappears within an hour after the sessions.

### 4.5 The Hair Mesotherapy Protocol

Hair mesotherapy is reported to require numerous sessions and be time‐consuming and costly [21]. There is no standard protocol for the interval period between the sessions, amount of product applied, or the number of courses. As a result of this study, we completed scalp mesotherapy over a 3‐month period, with one session per week for the first month (4 sessions in total) and then one session every 2 weeks for the following 2 months (another 4 sessions), totaling 8 sessions. 3 mL of the products were used, containing 1.5 mL of each. We believe that this hair mesotherapy protocol is efficacious for restoring AGA.

### 4.6 Trichoscopy and Trichoscan of AGA

The main indicative feature for trichoscopy of AGA is hair diameter diversity, which corresponds to the vellus transformation of more than 20% of the hairs [35]. Hair diameter variability, vellus hairs, and the peripilar sign are mentioned as being the trichoscopic indicators for the diagnosis of AGA [36]. In this study, hair diameter diversity was present in all patients. Following the hair mesotherapy protocol, a 30% reduction in this finding was observed, and it was statistically significant. Due to the prolongation of the kenogen phase in AGA, empty follicles appears [35]. We document empty follicles with a lower prevalence after the mesotherapy and conclude that diminishment of the empty follicles is a marker of successful therapy. Also, regrowing hairs present as upright vellus hairs appeared in 40% of our patients, which is a sign of promising results. The peripilar sign was reported with a comparable percentage to our study [36]. This feature did not disappear after mesotherapy. According to our trichoscopic features, we indicate hair mesotherapy as an encouraging method to treat AGA.

In a study comparing the efficacy of PRP and mesotherapy in AGA, a total of four different formulations were evaluated, including two different mesotherapy contents and two different PRP formulations [17]. The mesotherapy 1 content used in this study is the same as in our research. This mesotherapy content was reported to be more effective compared to the other mesotherapy formulation, with significant differences observed in hair count, thickness, and density [17]. Differences were also noted between the PRP brands in terms of outcomes, and it was demonstrated that both mesotherapy and PRP formulations significantly improved trichoscopic parameters in patients with AGA [17]. In another study, comparing the efficacy of the QR 678 with PRP in male androgenetic alopecia, videodermoscopic assessment showed significant improvement for terminal hair density and shaft diameter for QR 678 group [37]. In our study, Trichoscan results demonstrated that total hair count, hair density, hair thickness mean, density of terminal hairs and ratio of terminal hairs were significantly increased. The use of Trichoscan in our study provided an objective evaluation, enabling us to obtain more realistic results. Our findings were in line with the literature about biomimetic peptides' efficacy for AGA.

Studies conducted with hair mesotherapy products have some limitations, including a lack of negative controls and short follow‐up periods [38]. A Limitation of our study is not having any control group or follow‐up period.

Our findings align with previous studies showing that mesotherapy enhances the local bioavailability of active agents, thereby promoting hair regrowth while minimizing systemic side effects. While mesotherapy with minoxidil or dutasteride has shown efficacy, adverse effects such as edema, irritation, or injection‐site reactions have been reported. In contrast, our peptide‐ and micronutrient‐based approach was well‐tolerated.

Biomimetic peptides mimic the effects of growth factors such as VEGF, IGF‐1, and FGF, which play critical roles in angiogenesis, follicular stem cell activation, and prolongation of the anagen phase. These mechanisms are comparable to those of PRP, which has also demonstrated efficacy in AGA. The combination of biomimetic peptides with vitamins and amino acids may further support follicular metabolism and reduce miniaturization.

However, limitations of this study include its retrospective design, lack of a control group, and short follow‐up period. Future randomized controlled trials with larger populations and longer observation are required to confirm these results and optimize treatment protocols.

## 5 Conclusions

In this retrospective study, hair mesotherapy with biomimetic peptides, vitamins, and micronutrients demonstrated significant improvements in both trichoscopic and Trichoscan parameters in patients with AGA. The protocol was safe and well‐tolerated. These findings suggest that biomimetic peptide–based mesotherapy may represent a promising therapeutic option for AGA, but further large‐scale randomized controlled studies are needed to validate its efficacy, establish standardized protocols, and assess long‐term safety.

## Author Contributions

Ö.K.: conceptualization, methodology, software, validation, formal analysis, investigation, resources, data curation, writing – original draft preparation, writing – review and editing, visualization, supervision, project administration, funding acquisition; M.B.: conceptualization, software, writing – review and editing.

## Funding

The authors have nothing to report.

## Ethics Statement

The study was conducted in accordance with the Declaration of Helsinki and approved by the Erciyes University Medical and Health Sciences Clinical Research Board (Approval No: 2018/780).

## Consent

Informed consent was obtained from all subjects involved in the study.

## Conflicts of Interest

The authors declare no conflicts of interest.

## Data Availability

The data that support the findings of this study are available on request from the corresponding author. The data are not publicly available due to privacy or ethical restrictions.
